# Homemade blenderized tube feeding improves gut microbiome communities in children with enteral nutrition

**DOI:** 10.3389/fmicb.2023.1215236

**Published:** 2023-08-23

**Authors:** Sayaka Katagiri, Yujin Ohsugi, Takahiko Shiba, Kanako Yoshimi, Kazuharu Nakagawa, Yuki Nagasawa, Aritoshi Uchida, Anhao Liu, Peiya Lin, Yuta Tsukahara, Takanori Iwata, Haruka Tohara

**Affiliations:** ^1^Department of Periodontology, Graduate School of Medical and Dental Sciences, Tokyo Medical and Dental University (TMDU), Tokyo, Japan; ^2^Department of Oral Medicine, Infection, and Immunity, Harvard School of Dental Medicine, Boston, MA, United States; ^3^Department of Dysphagia Rehabilitation, Graduate School of Medical and Dental Sciences, Tokyo Medical and Dental University (TMDU), Tokyo, Japan

**Keywords:** enteral nutrition, blenderized tube feeding, gut microbiota, oral microbiota, tube feeding

## Abstract

Enteral nutrition for children is supplied through nasogastric or gastrostomy tubes. Diet not only influences nutritional intake but also interacts with the composition and function of the gut microbiota. Homemade blenderized tube feeding has been administered to children receiving enteral nutrition, in addition to ready-made tube feeding. The purpose of this study was to evaluate the oral/gut microbial communities in children receiving enteral nutrition with or without homemade blenderized tube feeding. Among a total of 30 children, 6 receiving mainly ready-made tube feeding (RTF) and 5 receiving mainly homemade blenderized tube feeding (HBTF) were analyzed in this study. Oral and gut microbiota community profiles were evaluated through 16S rRNA sequencing of saliva and fecal samples. The α-diversity representing the number of observed features, Shannon index, and Chao1 in the gut were significantly increased in HBTF only in the gut microbiome but not in the oral microbiome. In addition, the relative abundances of the phylum *Proteobacteria*, class *Gammaproteobacteria*, and genus *Escherichia-Shigella* were significantly low, whereas that of the genus *Ruminococcus* was significantly high in the gut of children with HBTF, indicating HBTF altered the gut microbial composition and reducing health risks. Metagenome prediction showed enrichment of carbon fixation pathways in prokaryotes at oral and gut microbiomes in children receiving HBTF. In addition, more complex network structures were observed in the oral cavity and gut in the HBTF group than in the RTF group. In conclusion, HBTF not only provides satisfaction and enjoyment during meals with the family but also alters the gut microbial composition to a healthy state.

## 1. Introduction

Diet not only influences nutrient intake but also interacts with the composition and function of the gut microbiota (Lindell et al., 2022). Owing to its essential role in the intestinal barrier, the gut microbiota is related to the immune status, cerebral function, and various metabolic pathways of the host (Mowat and Agace, 2014; Thaiss et al., 2016; Margolis et al., 2021). Low diversity of the gut microbiota increases the risk of health problems (Browne et al., 2017), especially during the developmental stage when energy absorption is crucial for the growth of the brain and lungs (Ronan et al., 2021). Moreover, microbial diversity affects the risk of diseases that persist throughout life, such as asthma and inflammatory bowel disease (Milani et al., 2017). However, this factor has not been considered in the dietary adjustment of children undergoing eternal nutrition. Therefore, clinical evidence is urgently required to improve the physical and mental conditions of these patients.

Some children with disabilities rely on enteral nutrition as their primary source of nutrition because of a variety of factors caused by their primary disease. Enteral nutrition is required if nutritional requirements cannot be met by oral ingestion (Bischoff et al., 2022). Gastrostomy or enterostomy is considered in patients expected to receive enteral nutrition for more than 6 weeks (Braegger et al., 2010). Enteral nutrition is classified based on its properties (standard, high concentration, low concentration, and semi-solidified) and the degree of degradation of the protein nitrogen source (semi-digestible, digestible, and component nutrition) and is used according to the patient’s condition. A blenderized diet can be administered through nasogastric or gastric tubes, in addition to ready-made nutritional formulas. This feeding technique is known as blenderized tube feeding (BTF), catering to the requests of families who wish their patients to consume the same food as the rest of the family. Additionally, it provides meals that consider each patient’s health condition and constitution, such as intolerance to tube feeding, allergies, and dietary restrictions based on beliefs (Bobo, 2016). A prospective cohort study of children receiving BTF or conventional formula tube feeding showed that BTF was associated with fewer gastrointestinal symptoms, such as nausea and vomiting, less abdominal pain and diarrhea, lower healthcare costs, and higher patient satisfaction (Hron et al., 2019). However, there is currently little scientific evidence regarding the use of BTF.

In addition, we previously reported an association between oral and gut microbiomes. Oral food intake affects oral and gut microbiomes in patients recovering from enteral nutrition (Katagiri et al., 2019). However, whether the contents of tube feeding affect the oral or gut microbiome in patients with dysphagia remains unclear. Thus, the main purpose of the present study was to evaluate the gut microbial communities in children receiving enteral nutrition with or without homemade BTF. Secondary, to evaluate whether homemade BTF alters the oral microbiome.

## 2. Materials and methods

### 2.1. Participants

The participants in this study belonged to the Mamakoi group, underwent either gastrostomy or enterostomy, and received a gastroduodenal diet or enteral nutritional supplements. “Mamakoi” is a Japanese group of parents of children with eating and swallowing disorders who need nutritional support. Currently, 281 members participate in the group, including medical professionals. The group is involved in activities that promote BTF and provide and exchange information among members. The child members of Mamakoi were chosen based on the following criteria: their primary diseases were under control, their oral intake was insufficient due to their primary diseases, and they were on tube feeding. Members treated with antibiotics for the preceding 6 months at the date of sample collection were excluded from the study. Saliva and fecal samples were collected from 25 of the 30 children recruited for this study.

Approximately 1 mL of unstimulated saliva was collected from each participant, using a sterile tube. Saliva was collected by direct spitting into a sterile tube or from the oral cavity with a sterile syringe. Participants were instructed to refrain from eating, drinking, oral care, and use of mouthwash for 1 h prior to saliva sample collection and to collect the samples before bedtime. Feces were collected using a sterile cotton swab and placed in a 1.5-mL microcentrifuge tube. The collected samples were stored overnight in a −20°C freezer immediately after collection in each household. The next day, the samples were transported to the laboratory in a cooler box with a refrigerant and stored in a freezer at −20°C for approximately 70 days until further processing and analysis.

After excluding one member who was administered antibiotics during the study period, the remaining 24 members were surveyed by a dentist. Information, such as age, sex, height, weight, primary disease, duration from onset, duration of tube feeding, nutritional products, amount of intake, and route of administration of enteral nutrition, were collected. The functional level of oral food and liquid intake was evaluated using the Functional Oral Intake Scale (FOIS), a 7-point scale (Crary et al., 2005), defined as follows: score 1: can eat nothing by mouth; score 2: tube-dependent with minimal attempts of food or liquid; score 3: tube-dependent with consistent oral intake of food or liquid; score 4: total oral diet of a single consistency; score 5: total oral diet with multiple consistencies, but requiring special preparation or compensations; score 6: total oral diet with multiple consistencies without special preparation, but with specific food limitations; score 7: total oral diet without restriction. The Japan Coma Scale (JCS) (Ohta et al., 1986), which is used to evaluate consciousness, comprises the following four categories: JCS0, alert; JCS1, not fully alert but awake without any stimuli; JCS2, arousable with stimulation; and JCS3, unarousable.

**Figure 1:**
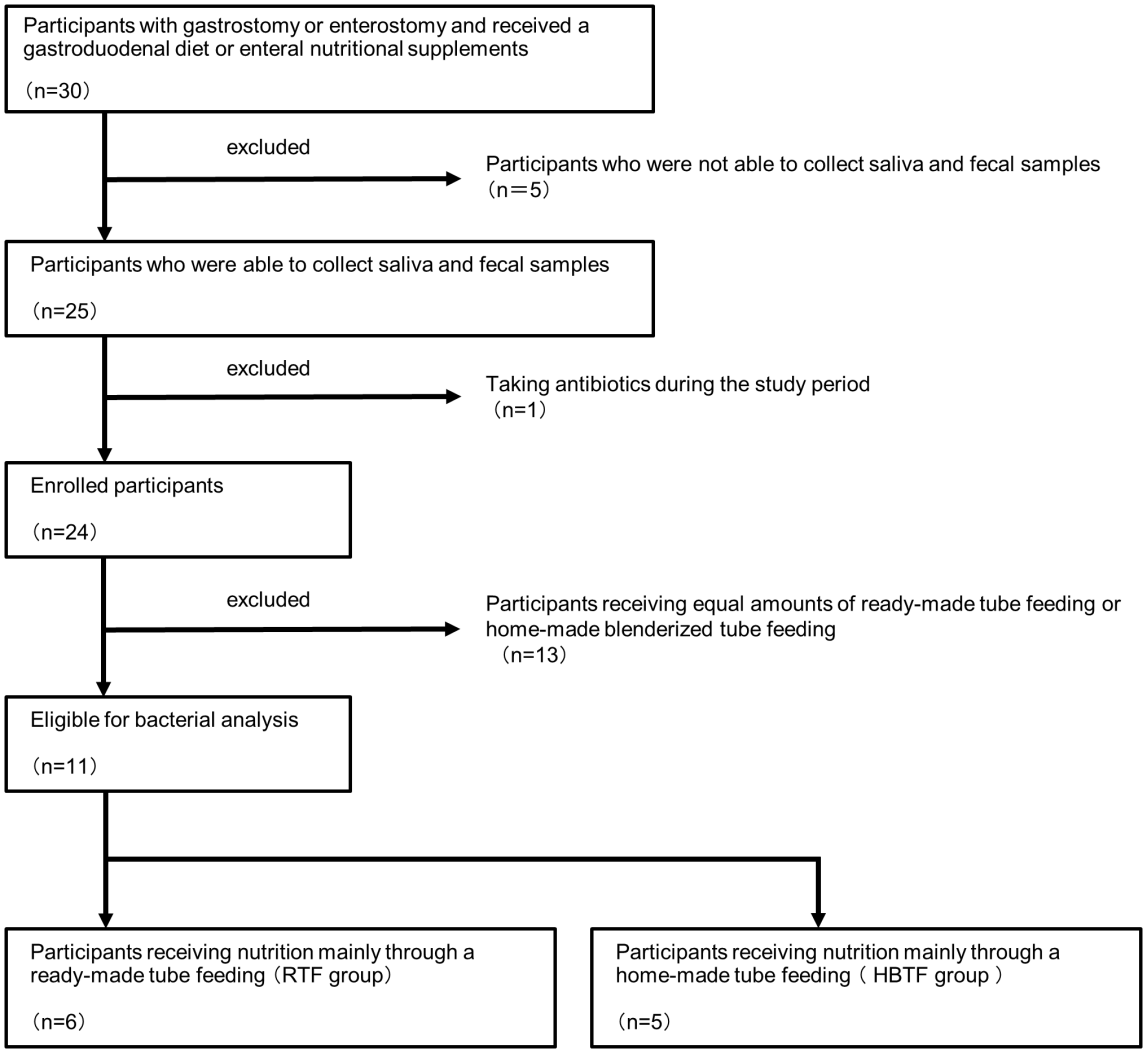
Trial profile.

Based on the obtained information, 13 members who received equivalent amounts of ready-made tube feeding (RTF) and homemade BTF (HBTF) were excluded, and 11 members were divided into two groups: those who received nutrition mainly through HBTF and those who received nutrition mainly through RTF (Figure 1). This was a human observational study, and the manuscript conforms to the guidelines for Strengthening the Reporting of Observational Studies in Epidemiology. The study was registered in UMIN (UMIN000050908). The study was conducted in accordance with the 1975 Declaration of Helsinki (revised in 2013) and was approved by the Ethics Committee of the hospital (D2019-095-01). Written informed consent for the study was obtained from all participants and their parents.

### 2.2. Evaluation of microbiome composition based on 16S rRNA gene sequences

DNA (average of 855 ± 293.61 ng, range: 70–6,530 ng) was extracted using GenCheck DNA Extraction Reagent (FASMAC, Kanagawa, Japan) from saliva and feces in the cotton swabs and quantified using Qubit 3.0 (Thermo Fisher Scientific, Waltham, MA, United States). Next, a multiplexed amplicon library targeting the V3–V4 region (341F/805R: 5′CCTACGGGNGGCWGCAG3′/5′GACTACHVGGGTATCTAATCC3′) was generated (Ohsugi et al., 2022). The first and second PCR was performed using an ExTaq HS DNA Polymerase kit (Takara Bio, Kusatsu, Japan). The first PCR conditions used were: 94°C for 2 min, followed by 20 cycles of 94°C for 30s, 50°C for 30s, and 72°C for 30s, and finally 72°C for 5 min. After purification of the products using an AMPure XP kit (Beckman Coulter Life Science, Indianapolis, IN, USA), the second PCR was performed using the following conditions: 94°C for 2 min, followed by 8 cycles of 94°C for 30 s, 60°C for 30s, and 72°C for 30s, and finally 72°C for 5 min. The library was sequenced using the MiSeq platform (Illumina, San Diego, CA, United States) to obtain 2 × 300 bp paired-end reads. Sequence data are available at the DNA Data Bank of Japan (DDBJ)^1^ database (DRA016128). The sequence data were processed and analyzed using the QIIME2 (Bolyen et al., 2019) (version 2022.2) custom pipeline. Primer trimming, quality check, and chimera filtering were performed using the q2-dada2 using the following parameters: –p-trim-left-*f* = 17, −p-trim-left-r = 21, −p-trunc-len-*f* = 270, and –p-trunc-len-r = 210. These parameters were determined by considering the following conditions: maintaining a 25-percentile sequencing quality score of 20 in forward reads, a minimum overlapping length of at least 16 bps, and displaying the maximum percent of input merged and non-chimeric (Seo-Young et al., 2021). All samples except one sample were subsampled to depth of 23,000 reads for only constructing rarefaction curve using the rarefaction.single command in mothur software, version 1.33.3 (Schloss et al., 2009). Taxonomic classification of amplicon sequence variants (ASVs) was performed using all sequence reads and the QIIME feature-classifier classify-sklearn based on SILVA 138 at 99% sequence similarity. Alpha diversities were calculated using Shannon index and Chao1 within the Qiime2 platform. Before performing the β-diversity analysis, the R package compositions, and zCompositions according to the CoDa_microbiome_tutorial,^2^ and ASVs with a mean read count of less than 1 across all samples were removed. The read abundance was converted into a centered log-ratio (clr; Gloor et al., 2017). Principal component analysis (PCA) was performed on clr-transformed values to visualize β-diversity (Weiler et al., 2018). We also performed hierarchical clustering using the hclust command with Euclidian distance and complete linkage based on the clr-transformed value (Weiler et al., 2018). Additionally, heatmaps were visualized based on the clr-transformed values using heatmap2.

PICRUSt2 plugin for QIIME2 (q2-picrust2 ver. 2021.2; Douglas et al., 2020) was employed to predict the functional abundances based on 16S rRNA gene sequencing data. Functional composition of the data was predicted based on the Kyoto Encyclopedia of Genes and Genome (KEGG) orthology database (Kanehisa et al., 2016). Co-occurrence coefficients at the genus level were calculated using the SparCC program (Friedman and Alm, 2012). Ten iterations were used to estimate the median correlation of each pairwise comparison, and each correlation was calculated by bootstrapping with 500 iterations (Milici et al., 2016). The parameters used for this analysis were as previously described, except for the SparCC values (Shiba et al., 2016; Watanabe et al., 2021). Taxon pairs with SparCC values ≥0.9 are considered to have a co-occurrence relationship with a positive correlation. The statistical significance thresholds were *p* > 0.05 and *q* > 0.1 using PseudoPvals in SparCC and Benjamini-Hochberg’s procedure, respectively. The networks were visualized using Cytoscape software v.2.8.

### 2.3. Statistical analysis

The characteristics between the RTF and HBTF groups were evaluated using unpaired *t*-test for age, body mass index (BMI), and duration of tube feeding, Fisher’s Exact Test for sex, and Mann–Whitney U test for JCS and FOIS. Statistical analyses were performed by SPSS version 28 (IBM Japan, Tokyo, Japan).

Permutational multivariate analysis of variance (PERMANOVA, 9,999 permutations, ‘adonis2’ function, Bray–Curtis dissimilarity) was performed to evaluate the significance of dissimilarity between the two groups using vegan R package (version 2.6.4) after removing ASVs with a mean read count of less than 1 across all samples. Differentially abundant taxa were identified using the analysis of the composition of microbiomes (ANCOM) R package (Mandal et al., 2015) (Third Release, 2019). Before ANCOM, a 10% prevalence filtering was applied to filter out any ASVs present in less than 10% of the samples within each dataset, as previously reported (Wallen, 2021; Nearing et al., 2022). For data processing, “feature_table = otu_data,” “sample_var = “SampleID,”” “group_var = NULL,” “out_cut = 0.05,” “zero_cut = 0.90,” “lib_cut = 1,000,” “neg_lb = FALSE” were inputted, indicating prevalence filter of 10% was applied. For the correction of the value of *p*, “p_adj_method = “BH”” and “alpha = 0.05” were inputted. W statistics greater than or equal to 60% of the total number of taxa tested were considered significant (>W_0.6_).

## 3. Results

### 3.1. Characteristics of the study population

Table 1 presents the characteristics of the study participants. Of the 11 participants, only one in the RTF group underwent an enterostomy, while the remaining 10 participants underwent gastrostomies. Primary diseases and types of nutritional supplements are listed in the table. No significant differences were observed in age (*p* = 0.478), sex (*p* = 0.455), Japan Coma Scale scores (*p* = 0.662), Functional Oral Intake Scale levels (*p* = 0.177), body mass index (*p* = 0.832), or duration of tube feeding (*p* = 0.131) between the two groups.

**Table 1:** Characteristics of the study population.

Group	No.	Age	Sex	JCS	FOIS	BMI	Primary disease
RTF	1	15	M	0	2	15.7	Crohn disease
	2	14	M	0	1	9.0	Trisomy18 syndrome
	3	14	M	0	1	12.9	Acute encephalopathy
	4	11	F	2	2	10.4	Mitochondrial disease
	5	11	M	0	1	14.0	Cacna1e mutation
	6	8	F	0	1	20.8	Holoprosencephaly
HBTF	1	10	M	0	2	12.0	Acute encephalopathy
	2	17	M	0	1	16.7	Mitochondrial encephalomyopathy
	3	15	M	0	2	13.6	Bacterial meningitis
	4	11	M	0	2	14.6	Cerebral palsy
	5	14	M	0	3	14.3	Cerebral palsy

Group	No.	Type of nutritional supplement	Route of nutrient administration	Duration of tube feeding (years)
RTF	1	RACOL-NF Semi solid®, ELENTAL®	Gastrostomy	13
	2	RACOL-NF Liquid®	Gastrostomy	13
	3	MA LACFIA®	Gastrostomy	7
	4	GLUSERNA®, ENEBO®, HBTF	Intestinal fistula	8
	5	ENORAS Liquid®	Gastrostomy	5
	6	KETONFORMULA®	Gastrostomy	8
HBTF	1	HBTF	Gastrostomy	6
	2	HBTF	Gastrostomy	2
	3	HBTF	Gastrostomy	7
	4	HBTF	Gastrostomy	9
	5	HBTF, RACOL-NF Semi solid®	Gastrostomy	6

RTF, ready-made tube feeding; HBTF, homemade blenderized tube feeding; JCS, Japan Coma Scale; FOIS, functional oral intake scale; BMI, body mass index.

### 3.2. Evaluation of oral and gut microbiome compositions based on 16S rRNA gene sequences

A total of 1,134,708 sequence reads were generated through 16S rRNA gene sequencing, with an average of 51,578 ± 2,951 (range: 6,459–72,027) reads per sample (Supplementary Table S1). The rarefaction curves exhibited a characteristic plateau for all samples included in this study, indicating that sufficient reads were obtained for the 16S rRNA gene analysis (Tian et al., 2019; Wang et al., 2019; Supplementary Figure S1). The percentages of unclassified reads were low (Supplementary Table S2).

**Figure 2:**
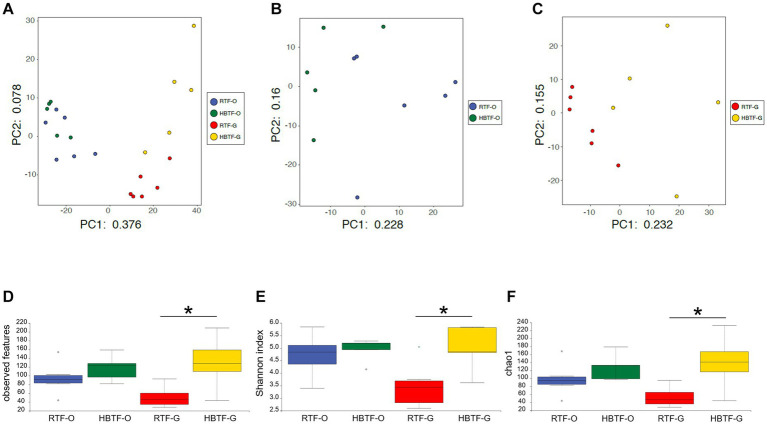
Evaluation of oral and gut microbiome compositions based on 16S rRNA gene sequences between RTF and HBTF participants (*n* = 6 and 5, respectively). PCA analysis **(A)** in the oral and gut microbiome between RTF and HBTF participants, **(B)** in the oral microbiome between RTF and HBTF participants, and **(C)** in the gut microbiome between RTF and HBTF participants. **(D)** Number of observed features, **(E)** Shannon index, **(F)** Chao1 among oral and gut microbiome between RTF and HBTF participants. RTF, ready-made tube feeding; HBTF, homemade blenderized tube feeding; RTF-O, oral microbiome in the participants with RTF; HBTF-O, oral microbiome in the participants with HBTF; RTF-G, gut microbiome in the participants with RTF; HBTF-G, gut microbiome in the participants with HBTF. *p < 0.05.

PCA revealed variations in the microbiome composition of saliva and feces, indicating that the microbial communities in the mouth and gut differed markedly (Figure 2A), as determined through PERMANOVA (*F* = 12.625, *R*^2^ = 0.38697, *p* = 0.0001). Oral microbial composition (Figure 2B) differed slightly but significantly between the two groups (PERMANOVA *F* = 1.7595, *R*^2^ = 0.16353, *p* = 0.041), whereas the gut microbiome showed a different composition (Figure 2C; PERMANOVA *F* = 2.7224, *R*^2^ = 0.23224, *p* = 0.0017). In addition, the number of observed features, Shannon index, and Chao1 in the gut microbiome were significantly increased in the HBTF group only in the gut microbiome (*p* < 0.05, unpaired *t*-test with Bonferroni correction); however, those in the oral microbiome were comparable between the two groups (Figures 2D–F).

**Figure 3:**
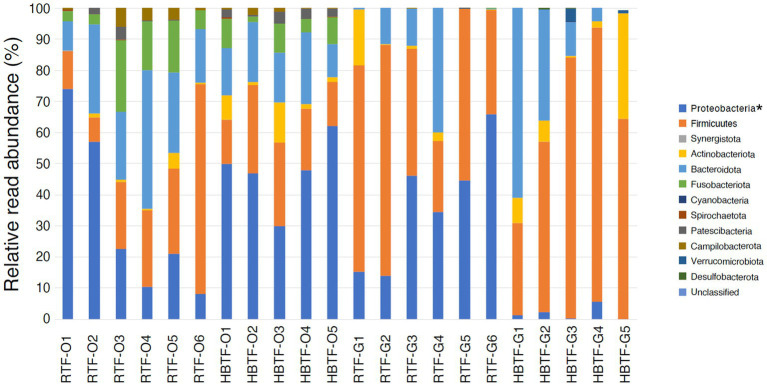
Evaluation of oral and gut microbiome compositions based on 16S rRNA gene sequences between RTF and HBTF participants at the phylum level (*n* = 6 and 5, respectively). * > W_0.7_ in gut microbial composition between RTF and HBTF participants. RTF, ready-made tube feeding; HBTF, homemade blenderized tube feeding; RTF-O, oral microbiome in the participants with RTF; HBTF-O, oral microbiome in the participants with HBTF; RTF-G, gut microbiome in the participants with RTF; HBTF-G, gut microbiome in the participants with HBTF.

**Figure 4:**
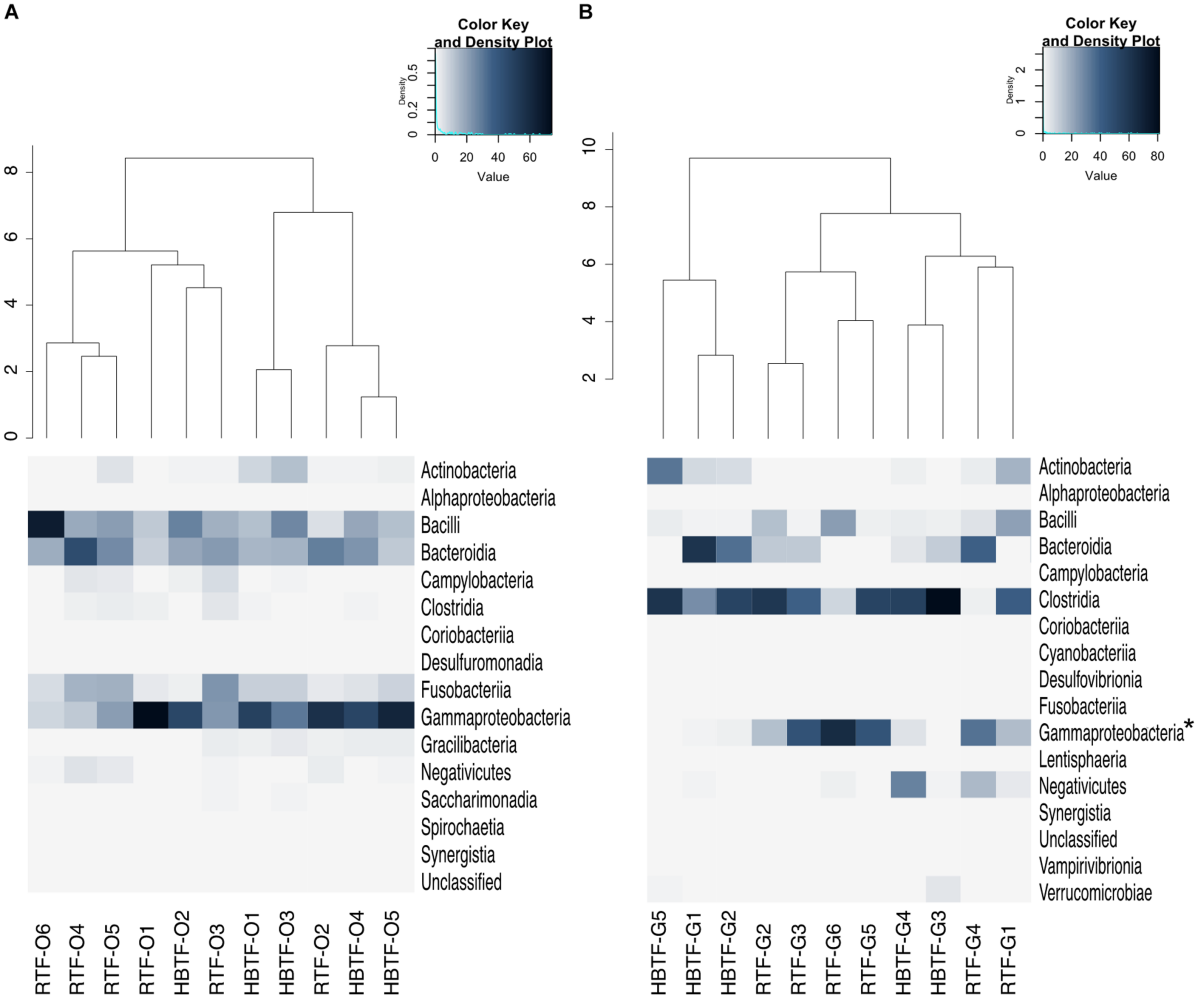
Evaluation of **(A)** oral and **(B)** gut microbiome compositions based on 16S rRNA gene sequences between RTF and HBTF participants at the class level (*n* = 6 and 5, respectively). Dendrogram and heatmap were constructed based on relative read abundance. * > W_0.7_ in gut microbial composition between RTF and HBTF participants. RTF, ready-made tube feeding; HBTF, homemade blenderized tube feeding; RTF-O, oral microbiome in the participants with RTF; HBTF-O, oral microbiome in the participants with HBTF; RTF-G, gut microbiome in the participants with RTF; HBTF-G, gut microbiome in the participants with HBTF.

**Figure 5:**
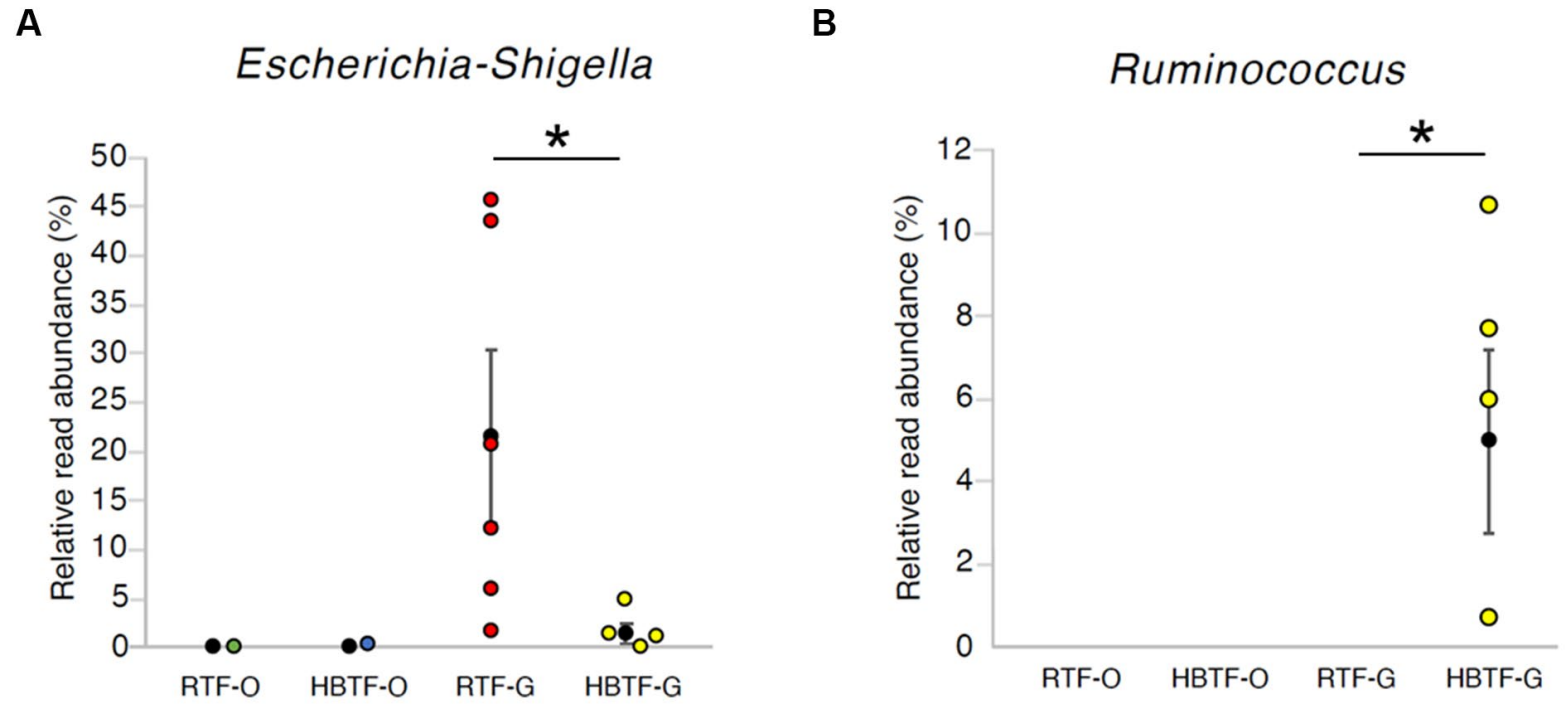
The relative read abundance in **(A)** genus *Escherichia-Shigella* and **(B)** genus *Ruminococcus*. * > W_0.6_ in gut microbial composition between RTF and HBTF participants. RTF, ready-made tube feeding; HBTF, homemade blenderized tube feeding; RTF-O, oral microbiome in the participants with RTF; HBTF-O, oral microbiome in the participants with HBTF; RTF-G, gut microbiome in the participants with RTF; HBTF-G, gut microbiome in the participants with HBTF.

No significant differences were observed in the relative abundance of the oral microbiome at the phylum level between the RTF and HBTF groups. The relative abundance of the phylum *Proteobacteria* was significantly low in the gut (>W_0.7_; relative abundance: RTF, 36.71; HBTF 1.97: −18.66-fold change) in the individuals receiving HBTF (Figure 3; Supplementary Table S3). In addition, the relative abundance of the class *Gammaproteobacteria* was significantly decreased (>W_0.7_; relative abundance; RTF 36.71; HBTF 1.90: −19.32-fold change) in the gut microbiome of participants under HBTF. The oral microbiome did not show a significant difference in composition between the RTF and HBTF groups at the class level (Figures 4A,B; Supplementary Table S4). No significant difference was observed in the oral or gut microbial composition between the two groups at the order (Supplementary Table S5) and family (Supplementary Table S6) levels. Although no significant differences in the oral microbiome composition were observed, the relative abundance of the genus *Escherichia-Shigella* was decreased (>W_0.6_; relative abundance: RTF 21.66; HBTF 1.50: −14.4-fold change; Figure 5A) in the gut of the HBTF group, whereas that of the genus *Ruminococcus* was increased (>W_0.6_; relative abundance; RTF 0.00; HBTF 4.98; Figure 5B). The relative abundance at the genus level is shown in Supplementary Table S7.

### 3.3. Metagenome prediction of the oral and gut microbiomes

**Figure 6:**
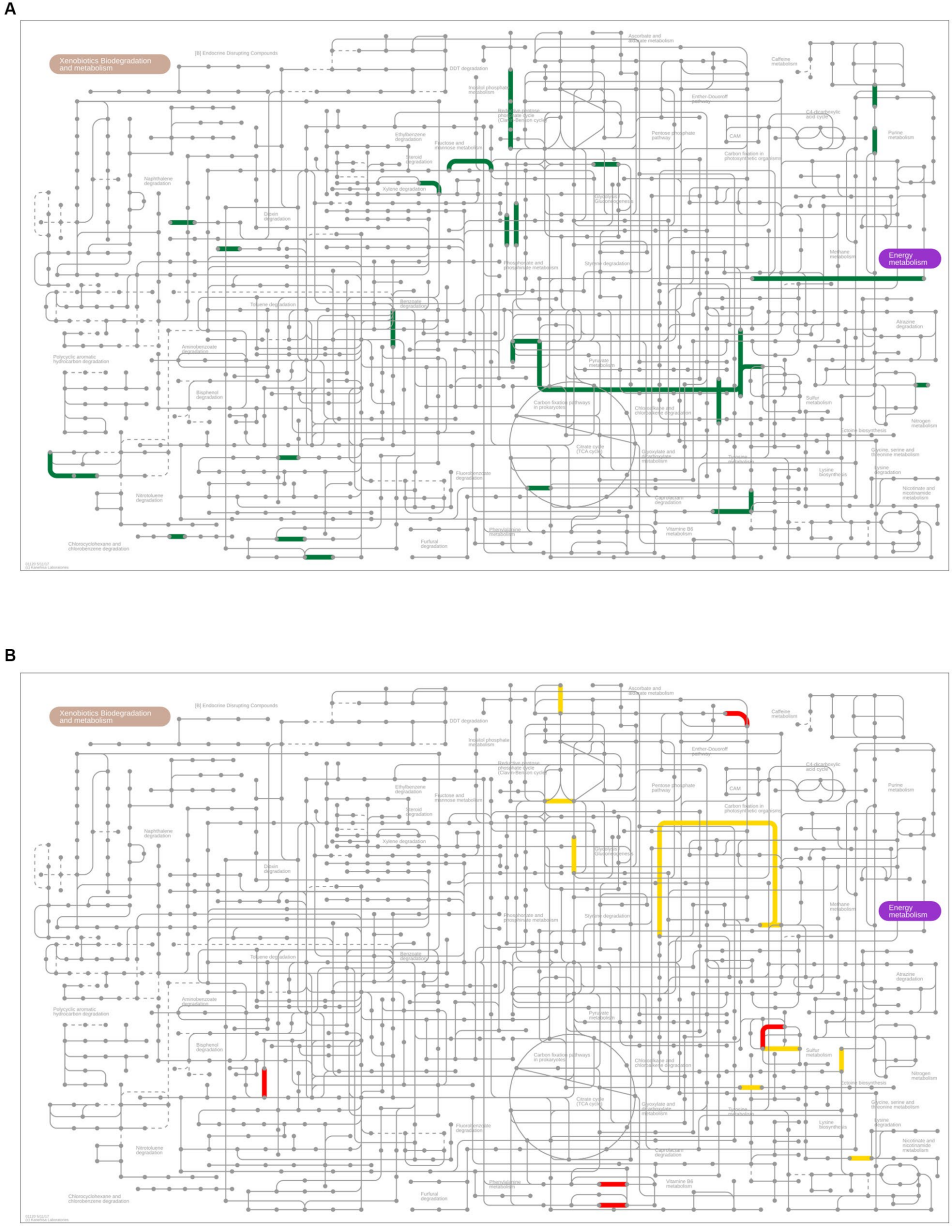
Metagenome prediction between RTF and HBTF groups (*n* = 6 and 5, respectively). **(A)** Upregulated genes (*p* < 0.01) predicted Kyoto Encyclopedia of Genes and Genome (KEGG) pathways in the oral microbiome of the HBTF group (green) in the microbial metabolism map. No predicted KEGG pathway was upregulated in the RTF group. **(B)** Predicted KEGG pathways of the upregulated genes (*p* < 0.01) in the gut microbiome of the RTF (red) or HBTF groups (yellow) in the microbial metabolism map. RTF, ready-made tube feeding; HBTF, homemade blenderized tube feeding.

PICRUSt2 analysis was performed to predict the relative abundance of gene functions in the oral and gut microbiomes. Although the oral microbial composition did not differ between the RTF and HBTF groups, 137 functional profiles (up, 136 profiles; down, one profile) were predicted to exhibit differences between the two groups (*p* < 0.01, unpaired *t*-test; Supplementary Table S8). Notably, sulfur and methane metabolisms and carbon fixation pathways in prokaryotes were significantly enriched in the HBTF group compared to those in the RTF group (Figure 6A). The gut microbiome showed 271 functional profiles (*p* < 0.01, unpaired *t*-test; up, 195 profiles; down, 76 profiles) by HBTF administration (Supplementary Table S9). Carbon fixation pathways in prokaryotes were also enriched in the gut microbiome of individuals with HBTF. Particularly, the reductive acetyl-CoA pathway (Wood-Ljungdahl pathway) was identified as a module in the carbon fixation pathways in prokaryotes enriched in the gut (Figure 6B).

### 3.4. Network analysis in the oral and gut microbiomes

**Figure 7:**
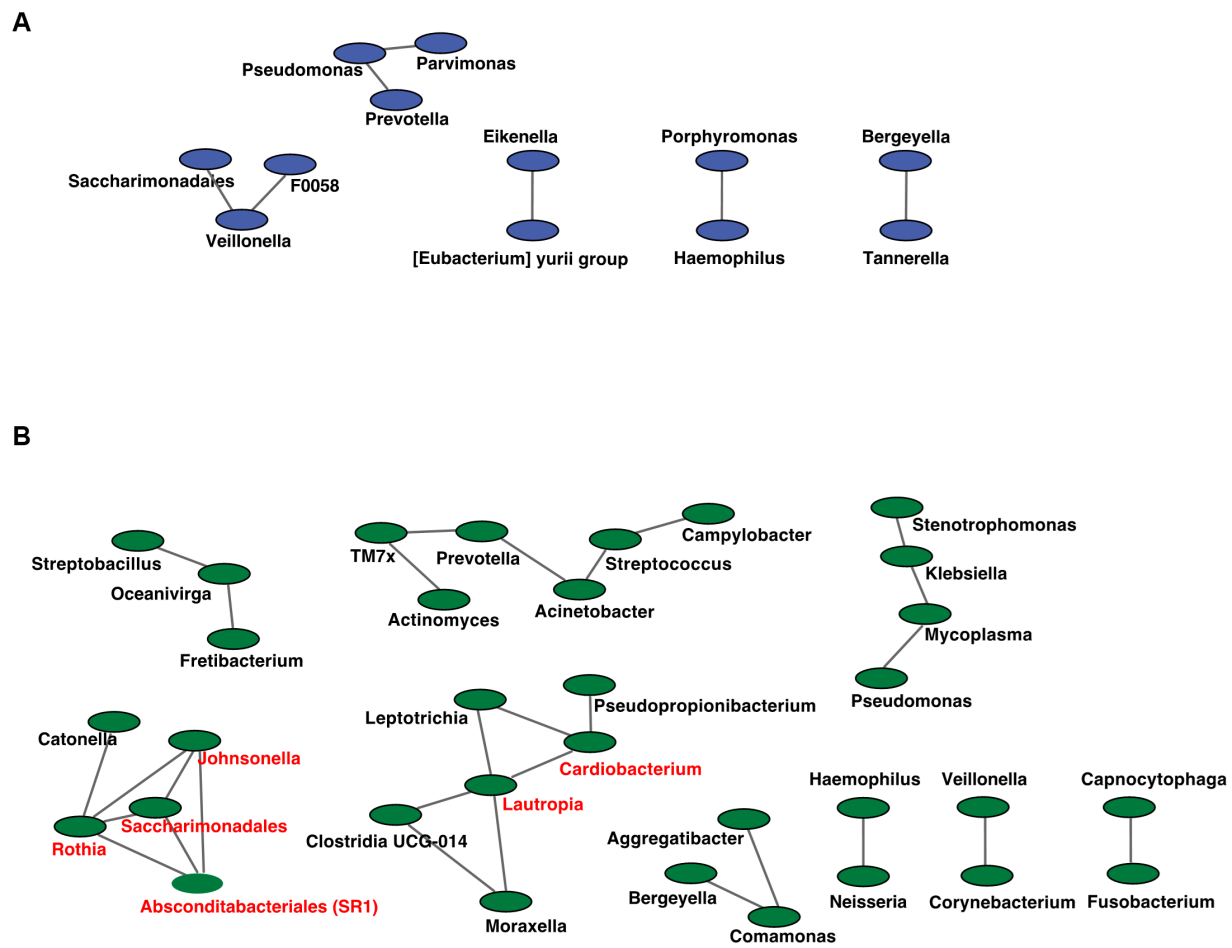
Co-occurrence network in the oral microbiome. All networks are shown with each genus and co-occurrence relationship indicated by a node and edges, respectively. Interacting core genus (showed co-occurrence with other genera ≥3) is indicated in red text. **(A)** RTF **(B)** HBTF. RTF, ready-made tube feeding; HBTF, homemade blenderized tube feeding; RTF-O, oral microbiome in the participants with RTF; HBTF-O, oral microbiome in the participants with HBTF; RTF-G, gut microbiome in the participants with RTF; HBTF-G, gut microbiome in the participants with HBTF.

We analyzed the co-occurrence relationships in the 16S rRNA gene profiles of each genus by constructing network structures in which each genus and co-occurring relationship were indicated by nodes and edges. The clustering coefficients of the oral microbiome were 0.000 and 0.217 for the RTF and HBTF groups, respectively. We identified 12 and 33 nodes in the RTF (Figure 7A) and HBTF (Figure 7B) groups, respectively. No statistically significant correlations were identified in this study. Any genus with three or more co-occurrences with other genera was classified as an “interacting core genus.” Although no interacting core genus was identified in the oral microbiome of the RTF group, six interacting core genera were observed in that of the HBTF group.

**Figure 8:**
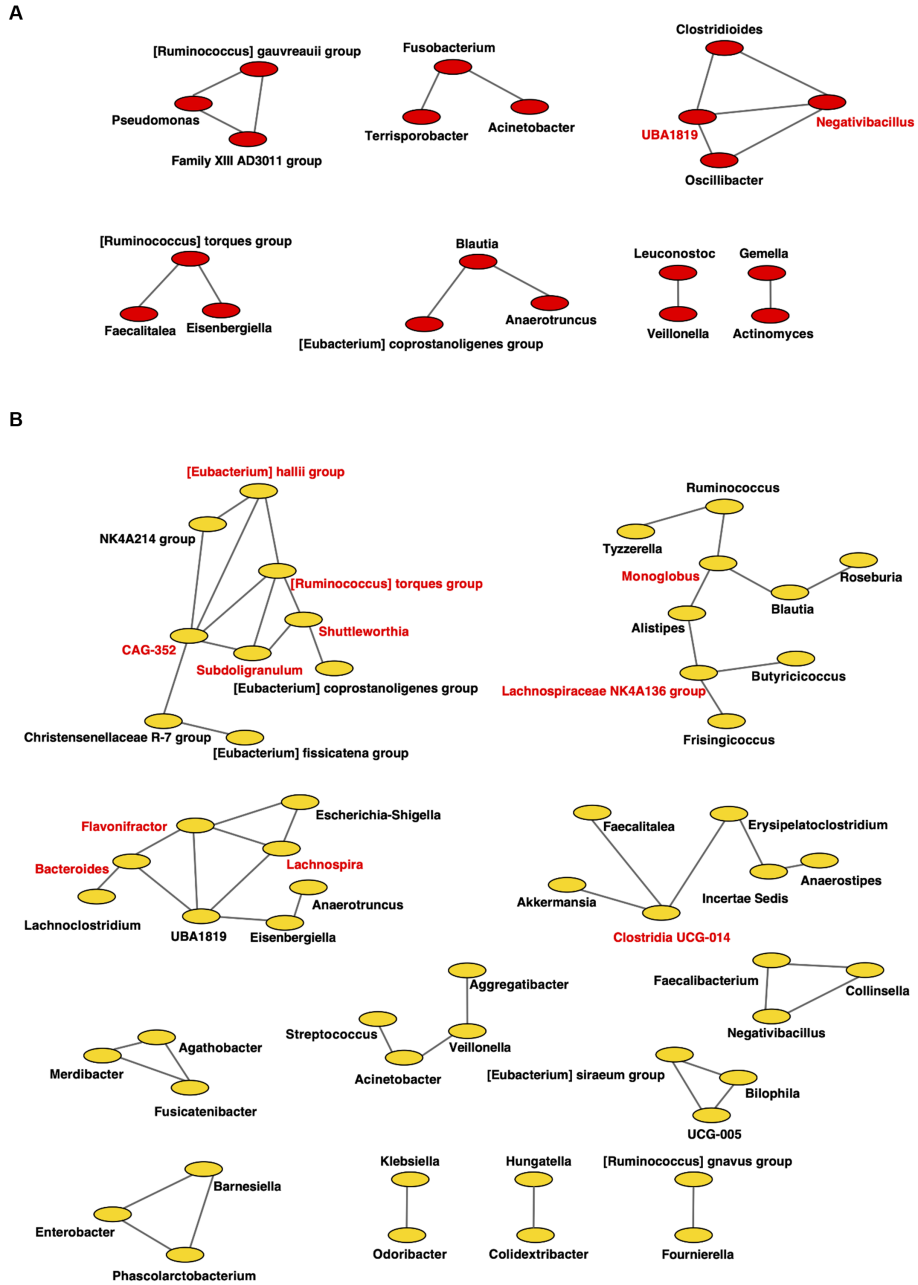
Co-occurrence network in the gut microbiome. All networks are shown with each genus and co-occurrence relationship indicated by a node and edges, respectively. Interacting core genus (showed co-occurrence with other genera ≥3) is indicated in red text. **(A)** RTF **(B)** HBTF. RTF, ready-made tube feeding; HBTF, homemade blenderized tube feeding; RTF-O, oral microbiome in the participants with RTF; HBTF-O, oral microbiome in the participants with HBTF; RTF-G, gut microbiome in the participants with RTF; HBTF-G, gut microbiome in the participants with HBTF.

For the gut microbiome, the clustering coefficients were 0.317 and 0.333 in individuals from RTF (Figure 8A) and HBTF (Figure 8B) groups, respectively. In addition, 20 and 55 nodes were identified in the RTF and HBTF groups, respectively. Only two genera were classified as interacting core genera in the RTF group, however, 11 interacting core genera were identified in the HBTF group.

## 4. Discussion

The current understanding of nutrition indicates that diet is important not only due to its nutritional benefits but also its effects on gut bacteria and other microorganisms. The extensive health benefits of these commensals include improved immunity, energy expenditure, drug metabolism, and cognitive function (Diaz Heijtz et al., 2011; Holmes et al., 2011). Most previous studies on HBTF have focused on its nutritional and medical values and physical properties, and only a few studies have examined its effect on the gut microflora (Vieira et al., 2018; Ojo et al., 2020a,b; Brekke et al., 2022). In this study, a significant increase in the diversity of the gut microflora, with no significant changes in the oral microbial diversity, was observed. The diversity of the gut microbiota is likely to have important effects on host immunity education during childhood. Low α-diversity and relative abundance of gut commensal bacterial genera, including *Bifidobacterium*, *Faecalibacterium*, *Ruminococcus*, and *Roseburia*, were associated with childhood respiratory diseases (Alcazar et al., 2022). Animal studies also suggested that germ-free mouse models had poorly developed gut-associated lymphoid tissue, lack mucosal T regulatory cell populations, and were prone to Th2 responses after sensitization compared to mice with diverse microbiota (Walter et al., 2020). Clinical evidence indicated that HBTF was well tolerated in gastrostomy-fed children, with improved upper gastrointestinal symptoms and enhanced quality of life (Hron et al., 2019; Batsis et al., 2020). In addition, HBTF increased the diversity of intestinal bacteria, similar to that observed in healthy children (Gallagher et al., 2018; Marchesi et al., 2022). In this study, significant differences in bacterial diversity were observed between the HBTF and RTF groups, suggesting that HBTF has broad effects on intestinal microbial ecology, which might contribute to its clinical benefits. Generally, the intestinal microbiota is more diverse than the oral microbiota (Katagiri et al., 2019). The results of the number of ASVs in this study were consistent with those previously reported, except gut microbiome in the RTF group (Kameoka et al., 2021; Chen et al., 2022). In this study, many participants are fed by tube for more than half of their lives. Thus, the unusual diversity in the gut microbiome in the RTF participants is considerable, although participants with HBTF showed higher diversity in the gut microbiome than in the oral microbiome.

Since the gut microbiota began to be considered as a new organ (Bäckhed et al., 2005), its characteristics have been defined as enterotypes and investigated in different populations and countries (Arumugam et al., 2011). Although the dominant enterotype in the Japanese population has changed to *Bacteroides*-rich (Takagi et al., 2022), *Ruminococcus* is still non-negligible due to its role in dietary fiber metabolism (La Reau and Suen, 2018). In adults, the increase in the abundance of *Ruminococcus* is a risk factor for various lifestyle diseases (Kurilshikov et al., 2019; Xu et al., 2022) due to its promoting effect on nutrient uptake (Boekhorst et al., 2022); however, the situation in children appears to be different. The increase of *Ruminococcus* was negatively correlated with childhood atopy and obesity (Tun et al., 2017), mainly due to the different metabolic pathways in the developmental stage. Moreover, a low abundance of *Ruminococcus* was observed in hand-foot-mouth disease (Guo et al., 2023), histamine intolerance (Sánchez-Pérez et al., 2022), and poor response to Bacillus Calmette-Guerin vaccination (Stražar et al., 2021), indicating its potential role in influencing the immune system during growth. In our study, *Ruminococcus* was present in four of the five fecal samples of the five participants receiving HBTF. The significant increase in gut *Ruminococcus* at the genus level in individuals under HBTF indicates the superiority of HBTF. Furthermore, our results showed a decrease in the abundance of the phylum *Proteobacteria*, class *Gammaproteobacteria*, and genus *Escherichia*, suggesting the reduction of symbiotic *Escherichia coli* as a type species at the genus level. Although symbiotic *E. coli* can provide vitamins when the host is malnourished (LeBlanc et al., 2013), considering the risk of being a potential pathogen through virulence factors (Starčič Erjavec and Žgur-Bertok, 2015; Khan et al., 2019), this benefit is less worthy. Symbiotic *E. coli* can exacerbate acute pancreatitis (Zheng et al., 2019), and its abundance increases in IgA nephropathy (Zhao et al., 2022), tuberculous meningitis (Li et al., 2022), inflammatory bowel disease (Khan et al., 2019), and Crohn’s disease (Nagayama et al., 2020). Hence, with the alteration in *Ruminococcus* abundance, HBTF can affect the composition of the microbiota in children under tube feeding, implying a health-risk-reduction effect.

Biological carbon fixation is the process by which living organisms convert inorganic carbon into organic compounds (Berg, 2011), and includes photosynthesis by plants and chemosynthesis by prokaryotes. Chemosynthesis also occurs in the gut microbiota. In our study, the Wood-Ljungdahl pathway (Ragsdale, 2008) was enhanced in the HBTF group. As the oldest carbon fixation pathway (Weiss et al., 2016), the Wood-Ljungdahl pathway is used by acetogenic and sulfate-reducing bacteria and methanogenic archaea, classified as hydrogenotrophic microbes (Smith et al., 2019). Acetate (mainly short-chain fatty acids), methane, and hydrogen sulfide are the main products and have an essential role in gut metabolism within the normal range. For instance, short-chain fatty acids positively affect type 2 diabetes (Canfora et al., 2015), methane is correlated with the α-diversity of the microbiota and irritable bowel syndrome (Chaudhary et al., 2018), and hydrogen sulfide plays an important role in mucosal immunity (Dilek et al., 2020). Additionally, as the hydrogen used in this metabolic process is a source of gut discomfort in adults and infants (Smith et al., 2019), HBTF benefits patients at multiple levels. However, this approach also activates methanogenesis in the oral microbiome of participants with HBTF, producing more methane in the oral cavity, which is a risk factor for halitosis and periodontitis (Lepp et al., 2004). The specific mechanism underlying the activation of the Wood-Ljungdahl pathway in the gut should be clarified further, and methanogenesis in the mouth should be examined and resolved in future investigations. However, metagenome prediction is only a prediction of functional genes based on the abundance of each taxon; therefore, metagenome analysis should be performed to obtain more robust evidence of the gene function in the microbiome.

In addition, the microbial network became more complex, as well as the increase of the number of networks in the oral cavity and gut of children receiving HBTF, indicating ecological changes in the oral/gut microbiota. Several studies have shown that the complexity and stability of microbial networks reflect the interactions among microorganisms. Without significant changes in the composition of gut bacteria, riboflavin allowed for a strengthened network of microbial interactions, resulting in increased butyrate production (Liu et al., 2023). Networks of oral bacteria also indicate the health status of the oral cavity. After fluoride treatment, the bacterial networks changed from simple to complex and tended to be in a healthy state, whereas no marked changes in the composition of the oral microorganisms were observed (Yang et al., 2023). The enhancement of network complexity in the oral and intestinal microflora provides new insights into the effects of HBTFs on the microbiota.

One of the issues of BTF, especially in homemade settings, is that the nutritional balance is not standardized, leading to nutritional deficiencies or an imbalanced nutritional diet in some patients (Vieira et al., 2018). A study comparing the effects of commercial formulas and BTF in adult patients with gastronomy undergoing concurrent chemoradiation therapy for head and neck cancer reported no difference in the nutritional status immediately after the initiation of BTF. However, the nutritional status worsened in the BTF group after 6 months (Papakostas et al., 2017). During the perioperative period or in patients with poor nutritional status, commercial formulas may be preferable for strict nutritional management due to deterioration in general health. The application of BTF is recommended for patients with stable general health while monitoring their nutritional status (Papakostas et al., 2017; Vieira et al., 2018). However, there is a risk of tube occlusion depending on the viscosity and texture of the BTF, and the use of a tube larger than 14 French gauge is recommended. Additionally, food should be blended until it is smooth, and the water content should be adjusted (Boullata et al., 2017). Clinical resources for BTF are available from the American Society for Parenteral and the European Society for Clinical Nutrition and Metabolism (Boullata et al., 2017; Bischoff et al., 2022), and frequent nutritional preparation is essential for each patient.

A survey of the family members of patients receiving tube feeding revealed that approximately half have adopted BTF, but only half have received instructions on BTF from medical professionals. The reasons for not introducing BTF often include a lack of knowledge (Schmitz et al., 2021). Education for healthcare professionals, the establishment of systems providing family guidance, and information on disease-specific BTF adjustments are necessary for introducing BTF. Moreover, BTF is often introduced to pediatric patients based on requests from their families, and most studies to date have focused on pediatric patients. In recent years, most adult patients in the United States have been administered BTF in combination with RTF. These patients experienced fewer instances of nausea, diarrhea, constipation, and other digestive problems than patients receiving only RTF (Hurt et al., 2015), which is similar to the results observed in pediatric patients (Hron et al., 2019). In older patients with significantly decreased swallowing function, a nasogastric tube or gastrostomy may be necessary to secure a nutritional route. In such cases, the introduction of BTF by the patients or their family may not only improve nutritional status and intestinal health, but also provide satisfaction and enjoyment during meals, which can contribute to an overall improvement in the quality of life.

A study compared the results of bacterial composition between the primers targeting the V1–V2, V1–V3, V3–V4, V4, V4–V5, V6–V8, and V7–V9 regions in the 16S rRNA gene using three different mock communities and human gut microbiota samples. The study revealed that the primer targeting the V3–V4 region is the most appropriate (Abellan-Schneyder et al., 2021). In another study, using a mock oral community, the primers targeting the V3–V4 and V4–V5 regions reportedly yielded higher result reproducibility (Teng et al., 2018). Therefore, in the present study, we selected the V3-V4 region to analyze the oral and gut microbiome. However, one limitation of this study is that the weights of the feces were not uniform, as fecal samples were collected using sterile cotton swabs, and the subsequent DNA extraction also involved the use of cotton swabs. In addition, samples were stored at −20°C, although ideal storage is at −80°C (Knight et al., 2018). We did not set positive and negative controls for DNA extraction; therefore, errors may have occurred during the extraction process (Hornung et al., 2019). Unfortunately, one fecal sample (HBTF1-G) only yielded 6,459 reads; however, as the rarefaction curve exhibited a plateau, we included the sample in our analysis. Furthermore, only male participants were enrolled in the HBTF group, although no statistically significant differences in sex were observed. There are a few reports on sex differences in the gut microbiome (Takagi et al., 2019; Hatayama et al., 2023). A previous study reported that the abundance of *Ruminococcus* was significantly higher in female participants than in male participants at the genus level (Takagi et al., 2019). In this study, the abundants of *Ruminococcus* was significantly increased in the gut of the HBTF group, and all individuals were male, indicating HBTF increased the abundance of *Ruminococcus* in the gut. Another study reported differences in the gut microbiome between men and women; *Fusobacterium*, *Megamonas*, *Megasphaera*, *Prevotella*, and *Sutterella* were more common in men, whereas *Alistipes*, *Bacteroides*, *Bifidobacterium*, *Odoribacter*, and *Ruthenibacterium* were more common in women (Hatayama et al., 2023). The number of participants was also limited. In the United States, almost 190 thousand children receive home enteral nutrition (Mundi et al., 2017). However, no reports have been published regarding the number of children receiving enteral nutrition in Japan. Moreover, Mamakoi is the largest group in Japan, comprising children and their families, to promote homemade BTF; therefore, it is difficult to increase the number of participants for this study.

In conclusion, this is the first study to show that HBTF increased the gut microbial diversity and altered the gut microbial composition and networks in enteral tube-fed children. Metagenome prediction indicated gene function was altered in both the oral and gut microbiota. However, further studies are required to establish stronger evidence for the promotion of HBTF.

## Data availability statement

The datasets presented in this study can be found in online repositories. The names of the repository/repositories and accession number(s) can be found in the article/Supplementary material.

## Ethics statement

The studies involving humans were approved by Ethics Committee of the Tokyo Medical and Dental University Hospital. The studies were conducted in accordance with the local legislation and institutional requirements. Written informed consent for participation in this study was provided by the participants’ legal guardians/next of kin.

## Author contributions

SK and YO performed most of the experiments and wrote the first draft of the manuscript. TS, KY, KN, YN, AU, AL, PL, YT, TI, and HT assisted in some studies and reviewed the manuscript. SK, TS, and KY supervised all the studies and the writing of the manuscript. All authors contributed to the article and approved the submitted version.

## Funding

This work was supported by the Japan Society for the Promotion of Science; 19K10221 and 22K10096 to HT, and Lotte Research Promotion Grant to SK.

## Conflict of interest

The authors declare that the research was conducted in the absence of any commercial or financial relationships that could be construed as a potential conflict of interest.

## Publisher’s note

All claims expressed in this article are solely those of the authors and do not necessarily represent those of their affiliated organizations, or those of the publisher, the editors and the reviewers. Any product that may be evaluated in this article, or claim that may be made by its manufacturer, is not guaranteed or endorsed by the publisher.

## Footnotes

^1^ http://www.ddbj.nig.ac.jp/

^2^ https://github.com/ggloor/CoDa_microbiome_tutorial
