# Supplementary figures and images for: Homemade blenderized tube feeding improves gut microbiome communities in children with enteral nutrition
Source: Front Microbiol. 2023 Aug 23;14:1215236. doi: 10.3389/fmicb.2023.1215236 (PMC10482415; doi:10.3389/fmicb.2023.1215236)

Supplementary Figure S1

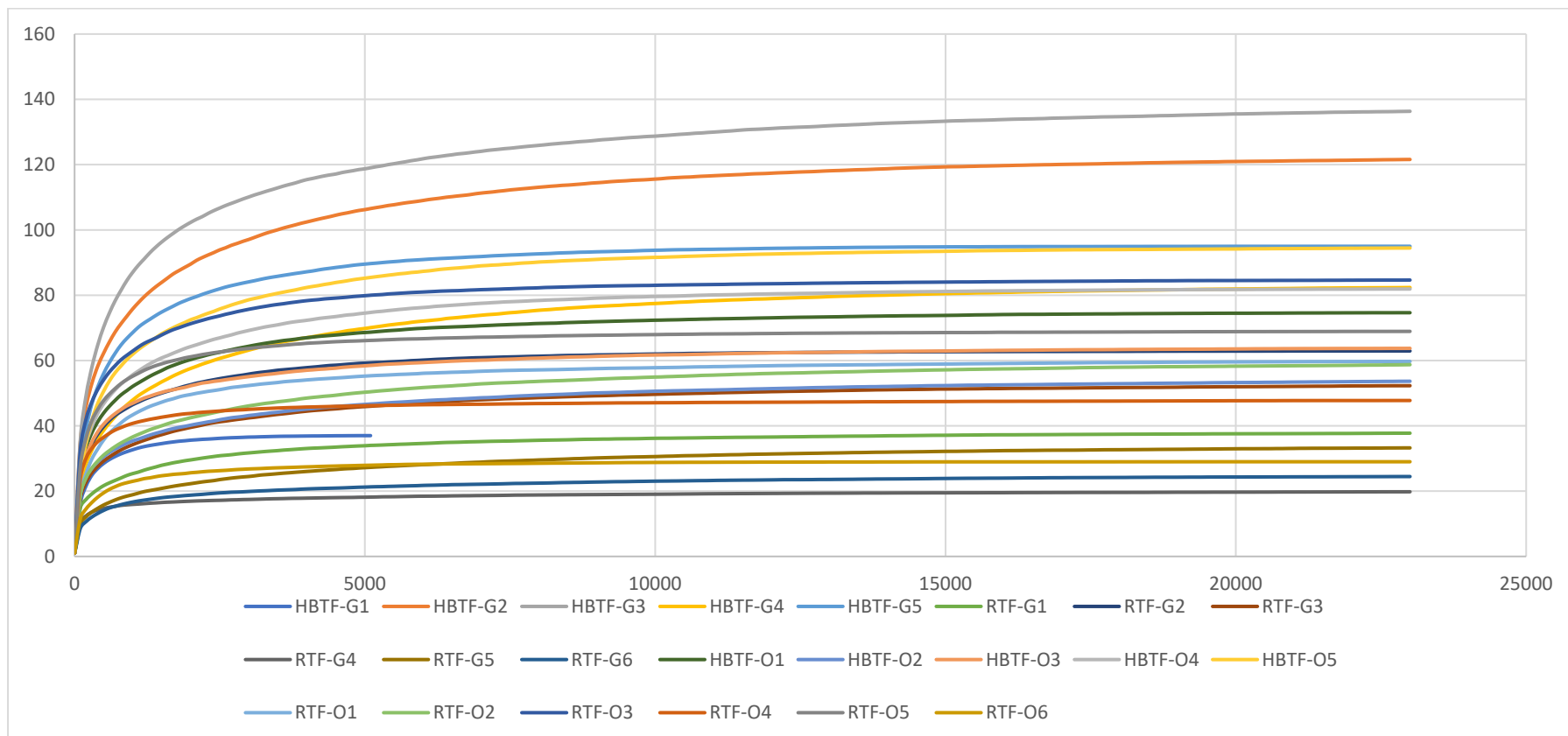

Supplement: Supplementary file 1 [file Image_1.pdf]
